# Characteristics of chest pain and its acute management in a low-middle income country: analysis of emergency department surveillance data from Pakistan

**DOI:** 10.1186/1471-227X-15-S2-S13

**Published:** 2015-12-11

**Authors:** Nino Paichadze, Badar Afzal, Nukhba Zia, Rakshinda Mujeeb, Muhammad Mujeeb Khan, Junaid A Razzak

**Affiliations:** 1 Johns Hopkins International Injury Research Unit, Department of International Health, Johns Hopkins Bloomberg School of Public Health, Baltimore, Maryland, USA; 2 Department of Emergency Medicine, Aga Khan University, Karachi, Pakistan; 3 Benazir Bhutto Hospital, Rawalpindi, Pakistan; 4 Department of Emergency Medicine, John Hopkins School of Medicine, Baltimore, Maryland, USA; 5 The author was affiliated with the Department of Emergency Medicine, Aga Khan University, Karachi, Pakistan at the time when study was conducted

**Keywords:** Emergency departments, chest pain, resources, Pakistan

## Abstract

**Background:**

Chest pain is one of the most frequent causes of emergency department (ED) visits in high-income countries. Little is known about chest pain patients presenting to EDs of low- and middle-income countries (LMICs). The objective of this study was to describe the characteristics of chest pain patients presenting to emergency departments (EDs) of Pakistan and to determine the utilization of ED resources in the management of chest pain patients and their outcomes.

**Methods:**

This study used pilot active surveillance data from seven major EDs in Pakistan. Data were collected on all patients presenting to the EDs of the participating sites to seek emergency care for chest pain.

**Results:**

A total of 20,435 patients were admitted to the EDs with chest pain. The majority were males (M 60%, F 40%) and the mean age was 42 years (SD+/- 14). The great majority (97%, n = 19,164) of patients were admitted to the EDs of public hospitals compared to private hospitals and only 3% arrived by ambulance. Electrocardiograms (ECGs) were used in more than half of all chest pain patients (55%, n = 10,890) while cardiac enzymes were performed in less than 5% of cases. Chest X-rays were the most frequently performed radiological procedure (21%, n = 4,135); more than half of the admitted chest pain patients were discharged from the EDs and less than 1% died in the ED.

**Conclusion:**

Chest pain is a common presenting complaint in EDs in Pakistan. The majority received an ECG and the use of diagnostic testing, such as cardiac enzymes, is quite uncommon.

## Background

Chest pain is a frequently occurring symptom affecting 20-40% of the general population worldwide [1-3]. It is responsible for more than 8 million visits to emergency departments (EDs) in the United States each year, making it the second most frequent cause of emergency visits [4] after stomach and abdominal pain [5]. Each year in England and Wales, approximately 15 million people visit EDs and 2.4% of attendances - representing 360,000 patient visits - are because of chest pain [6]. In the United States and Europe, up to 5% of visits to emergency departments are due to chest pain [7,8]. The burden of cardiovascular disease in low-and middle-income countries (LMICs) has gained increased attention [9-11], though population-based data on the prevalence of chest pain in developing countries is lacking [12].

Chest pain can be cardiac (angina) as well as non-cardiac in origin [13-15]. Patients present with a wide spectrum of signs and symptoms reflecting several potential etiologies of chest pain including life-threatening, urgent conditions such as: myocardial infarction (MI), pulmonary embolism, or aortic dissection; and non-urgent conditions such as: musculoskeletal pain, gastro-esophageal reflux disease (GERD), pericarditis, or others [16]. Chest pain is the most common presenting complaint of an MI [17].

Studies identifying characteristics of chest pain are mostly from developed countries, hence the data on global prevalence of chest pain in developing countries is scant [12]. Examining the prevalence of chest pain - both angina (cardiac) and non-anginal (non-cardiac) - and its characteristics in LMICs is critical for developing targeted interventions for the management of chest pain patients in EDs and potentially stemming an epidemic of premature coronary deaths [9].

The objective of this paper is to look at characteristics of chest pain patients presenting to EDs of Pakistan and to determine the utilization of ED resources in the management of chest pain patients and their outcomes.

## Methods

### Study setting and population

The Pakistan National Emergency Departments surveillance (Pak-NEDS) was an active pilot surveillance conducted from November 2010 - March 2011 in seven major emergency departments in Pakistan: Aga Khan University (AKU) and Jinnah Post-graduate Medical Center in Karachi; Benazir Bhutto Hospital in Rawalpindi; Lady Reading Hospital in Peshawar; Mayo Hospital in Lahore; Sandeman Provincial Hospital in Quetta; and Shifa International Hospital in Islamabad. All the sites were tertiary care urban centers. The AKU and the Shifa International Hospital are private hospitals while the rest are public hospitals. AKU was the main coordinating center for the study. Ethical approval for the study was taken from all participating sites. Data was collected on males and females of all age groups presenting to the emergency departments of the participating sites to seek emergency care for various medical and surgical conditions including chest pain. The total number of patients enrolled during the study period was 274,436. Chest pain as the major complaint and as the second and third presenting symptom was widely distributed among admitted patients. In this paper, we analysed only those who were admitted to the emergency departments with chest pain as a chief complaint.

### Data collection tool and team

A one-page standardized tool was developed based on an ambulatory care survey tool from the Centers for Disease Control and Prevention, USA and previous surveillance work done in Pakistan [18]. The tool had questions related to patient demographics like age, gender, ethnicity; presenting complaints; treatment and management provided in the emergency department; provisional diagnosis; and disposition from the emergency department. Data collectors were specifically hired and trained for this study and they worked in three shifts giving twenty-four hour coverage. Data collection was done through patient/next of kin interviews and review of emergency department records. Hard copies of the data collection tool were sent to the coordinating center at the AKU.

### Data analysis

Data was entered at AKU using Epi Info™ version 3.3.2. All data were tabulated using Stata, version 12 (Stata Corp, College Park, TX) and SPSS version 20 (IBM Corp, Armonk, NY) and used simple descriptive analytic methods. Available data on admitted chest pain patients was classified using simple, one-way, two-way, and complex tabulation statistical methods; frequency distribution and percentage calculations were reported for categorical variables. During the Pak-NEDS study period, 20,435 patients were admitted to the EDs with chest pain as a major complaint. Only up to 1% (n = 2,907) of all patients were admitted to the ED with other major complaints where chest pain was noted as the second (n = 2,452, 0.89%) or third (n = 455, 0.17%) additional presenting complaint, respectively. This number was considered negligible and thus the analysis only focused on patients who were admitted to the ED with chest pain as the chief complaint.

Considering differences in the presentation and the clinical course of chest pain between the adult and pediatric populations, we focused only on adult patients, between the ages of 18 and 90 years old (n = 19,752); correspondingly, patients younger than 18 years (n = 670, 3%) and older than 90 years (90-98 age group, n = 13, 0.06%) were excluded from further analysis. This decreased the total sample size to 19,752 patients. Age distribution was studied in the following main groups (in years): 18-30, 31-45, 46-60, 61-75, 75-90, and 81-90. For the multiple-response variables such as physical examination, diagnostic and screening services, conducted procedures, and visit disposition, only single, all available response options were considered in the analysis. For the variables with missing data, the percentage of missing data is presented.

## Results

**Figure 1:**
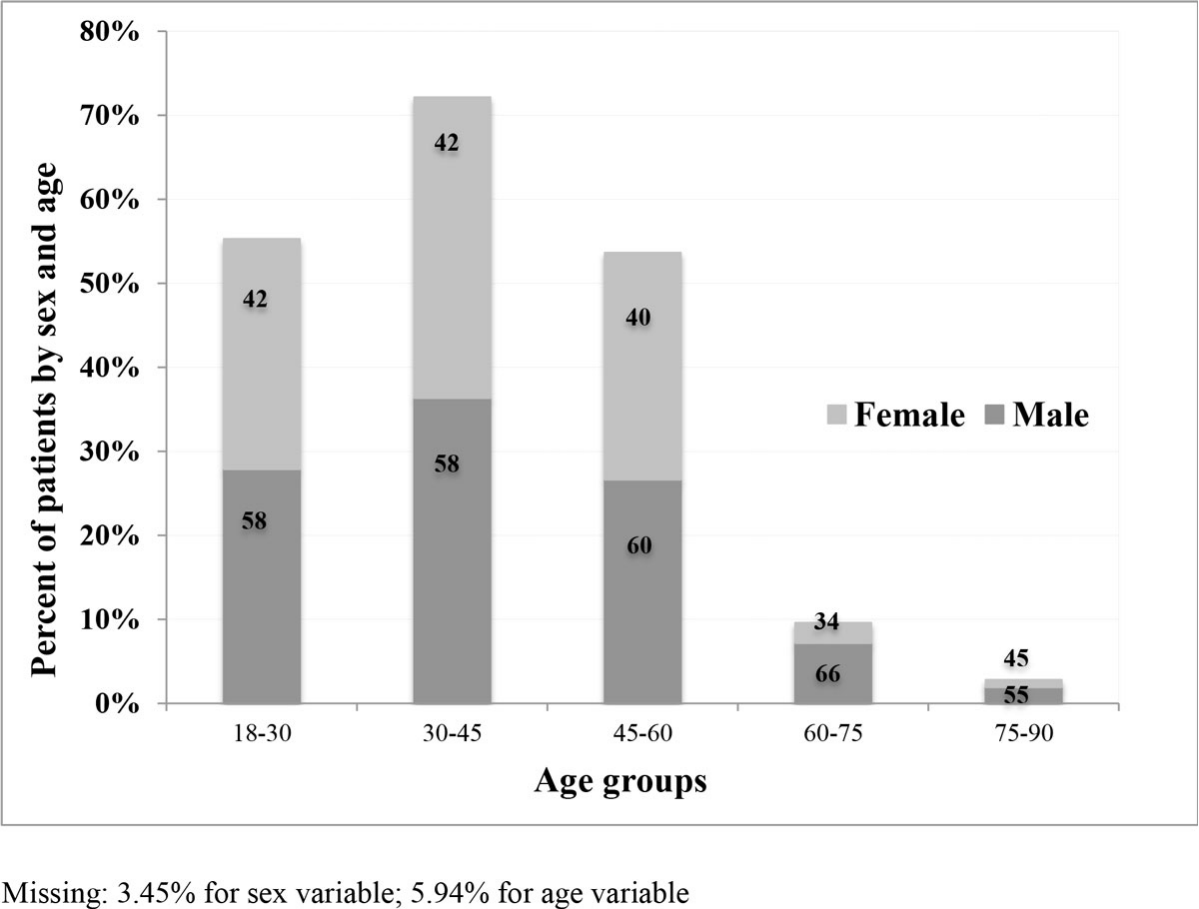
**Distribution of chest pain patients by age groups and sex, n = 19,752**.

Of the 19,752 patients, 58% (n = 11,419) were males and 39% (n = 7,651) were females. Almost 35% (n = 6,752) of all patients admitted with chest pain symptoms were in the 30-45 age group and less than 2% (n = 360) were in the 75-90 age group. The mean age of all admitted patients was 42 years (SD ±14). Age distribution as well as the mean age was similar among men and female (male: 42.14 ±13.67 years, female: 41.53 ±16.58 years) (Figure 1 and Table 1).

**Table 1:** Main characteristics of chest pain patients (n = 19,752)

History of visit n (%)		
**Sex**	Male	11,028 (60)
	Female	7,515 (40)

**Age**	18-30	5,181 (27.9)
	31-45	6,752 (36.3)
	46-60	4,949 (26.6)
	61-75	1,336 (7.2)
	75-90	360 (1.9)

**Mode of arrival at EDs**	Ambulance	585 (2.9)
	Non-ambulance	18,150 (91.9)

**Hospital**	Public	19,164 (97.0)
	Private	588 (3.0)

**Treated for chest pain in last 72 hours**	Male	1,888 (54.4)
	Female	1,546 (44.5)

**Episode of care**	First visit	16,532 (83.7)
	Follow-up visit	643 (3.2)
	Don't know	85 (0.4)

**Number of ED visits within last 12 months**	0	6,072 (30.7)
	1	3,750 (18.9)
	2	1,198 (6)
	3	540 (2.7)
	4-10	518 (2.6)
	Don't know	173 (0.9)

Missing: 5.13% for the mode of arrival variable; 3.55% for treatment in last 72 hours variable; 18.04% for discharged in last 7 days variable; 12.62% for episode of care variable; 37.89% for number of ED visits last year variable; 26.96% for triage variable

Nearly 97% (n = 19,164) of all chest pain patients were admitted to the ED of public hospitals and since the number of patients admitted to private hospitals was low (3%, n = 588), the analysis did not stratify results by hospital type. Less than 3% (n = 585) were delivered to the hospital by ambulance and 92% used other modes of transportation. Almost 18% (n = 3,471) of all admitted chest pain patients had been treated for chest pain in the last 72 hours before their current visit to the emergency department. For the majority of patients (84%, n = 16,532), this was the first episode of care for chest pain, and for 31% (n = 6,072), this was the first visit to the ED within the last 12 months (Table 1).

Review of the performed diagnostic and screening services showed that electrocardiograms (ECGs) were performed for more than half (55%, n = 10,890) of patients. Chest X-rays were the most frequently performed diagnostic test and were used in up to 21% (n = 4,135) of patients; cardiac enzymes were measured in only 5% (n = 1,010), with as low as 1% (n = 224) measured among females (Table 2).

**Table 2:** Imaging and disposition distribution based on gender and age groups (n = 19,752)

Sex	Imaging and Tests	Age groups					Total
			
		18-30	31-45	46-60	61-75	76-90	
Males	Cardiac enzymes	28(0.9)	296(7.5)	380(12.5)	76(8.7)	6(3.0)	786(7.1)
	ECG	1,357(44.9)	2,488(63.0)	2,052(68.8)	618(70.3)	134(67.3)	6,649(60.3)
	X-Ray	795(26.3)	849(21.5)	599(20.1)	188(21.4)	49(24.6)	2,480(22.5)
	
	Total males in each age group	3,021	3,948	2,981	879	199	11,028
Females	Cardiac enzymes	13(0.6)	69(2.5)	103(5.4)	27(6.0)	12(7.5)	224(3.0)
	ECG	943(44.5)	1,538(55.7)	1,312(68.2)	325(72.3)	122(76.3)	4,240(57.2)
	X-Ray	536(25.3)	618(22.4)	382(19.9)	82(18.3)	37(23.1)	1,655(22.3)
	
	Total females in each age group	2,119	2,763	1,924	449	160	7,415

**Sex**	**Disposition**	**Age groups**					**Total**
			
		**18-30**	**31-45**	**46-60**	**61-75**	**76-90**	

Males	Discharged	1,674(55.1)	2,275(57.6)	1,503(50.4)	362(41.2)	83(41.7)	5,897(53.5)
	Admitted	327(10.8)	580(14.7)	736(24.7)	273(31.1)	64(31.2)	1,980(18.0)
	Died	11(0.4)	21(0.5)	44(1.5)	26(3.0)	2(1.0)	104(0.9)
	
	Total males in each age group	3,021	3,948	2,981	879	199	11,028

Females	Discharged	1,084(51.2)	1,520(55.0)	941(48.9)	196(43.6)	69(43.1)	3,810(51.4)
	Admitted	231(10.9)	331(12.0)	449(23.2)	119(26.5)	48(30.0)	1,178(15.9)
	Died	9(0.4)	7(0.3)	22(1.1)	9(2.0)	6(3.8)	53(0.7)
	
	Total females in each age group	2,119	2,763	1,924	449	160	7,415

Overall, 16% (n = 3,158) of patients with chest pain were admitted to the hospitals, slightly higher for males compared to females (17% vs. 15%). A total of 157 (0.8%) of patients with chest pain died in the emergency department. The mortality rate was 25% higher for males compared to females (0.9% vs. 0.7%, p-value = 0.00).

## Discussion

This study looked at the characteristics of chest pain patients presenting at selected EDs in Pakistan. The analysis showed that more than 7% of all admitted patients had a complaint of chest pain. This finding is consistent with the numbers cited in published sources, which show that the prevalence of chest pain patients in EDs ranges between 2.4% and 20% in the United States, UK, and Europe [1,4,7,19]. Unfortunately, available literature on the burden of chest pain is limited to developed country settings only. Most literature from LMICs describes the prevalence, patterns, and risk factors of coronary heart disease (CHD) and MI [20-22], but information on chest pain as a symptom is scarce.

Patients presenting with chest pain were found to be young, with a mean age of 42 years and more likely to be males. This trend is comparable with other reports showing that patients with an MI from LMICs were younger than patients from high-income countries (HICs) [23-26][27]. An overall younger population, differences in risk factors, and socioeconomic disparities between populations in HICs and LMICs [24] could explain the higher prevalence of CHD in relatively young patients in Pakistan [28].

Pakistan has variable ambulance service quality with uneven coverage [29]. The findings of this study are in line with the already available evidence: most of the patients presented to the EDs used other means of transportation than ambulance service, which highlights the lack of pre-hospital care in Pakistan and thus may be a contributing factor to delayed presentation to the ED. Distance to facility was also found to be an important factor leading to delay in presentation, prolonging total time until treatment and resulting in worse outcomes in patients [30]. However, these variables were not recorded in this study.

Type and duration of chest pain, findings on the ECG and chest x-ray, and in some cases biomarkers are primary modalities used for triaging patients with chest pain in the emergency department. ECG is considered critical for decision-making; in many high-income settings, ECG done within 10-15 minutes of presentation to emergency department is considered one of the indicators for quality of care in chest pain management in the emergency department. In this study, only 55% of patients with chest pain had an ECG in the emergency department, with a higher rate shown in older patients. Additionally, biomarkers were rarely used in the emergency department, perhaps due to non-availability of the tests in the emergency department.

Less than 20% of all patients with chest pain were admitted to the hospital. This is a relatively low number of admission compared to findings in the US (35%) [31], but comparable to findings in the UK (25%) [6]. As previous research shows, only 10-15% of patients with chest discomfort have an acute MI, and most chest pain patients do not have significant disease [32-34]. Nevertheless, evidence from developed countries illustrates that physicians often fail to correctly diagnose an MI in patients with atypical signs and symptoms with as many as 4-13% of patients with an MI being discharged erroneously from the ED [32-34]. Because of this failure in the traditional approach to patients with chest pain, EDs in most developed countries started implementing chest pain units (CPUs) with designated resources of personnel, protocols, space, and equipment for patients presenting with chest pain [32]. Admission rates in Pakistan represent multiple factors working together, such as the prevalence of disease in the population, training and clinical practice of the providers, availability of resources such as hospital beds, and the preference of patients. Most of the care in our study was provided through government-run public hospitals. These hospital emergency departments are extremely busy with an average daily ED census between 400-1000. With such a high volume of patients, practitioners are likely to focus on patients with obvious findings on a clinical exam or ECGs. It is also not clear how many of these patients had a follow-up arranged during their visit to the emergency department and how many actually had an appropriate follow-up.

## Limitations

The Pak-NEDS study has several limitations, some of which were mentioned above. Due to a lack of a large amount of clinical information, analyses did not explore in detail: the characteristics of chest pain (duration of the symptom before visiting ED, character and site of pain, precipitating factors, etc.); risk factors and patient history (smoking status, diabetes, systolic blood pressure, etc.); the results of conducted screening and diagnostic tests; provisional diagnoses; or conducted treatment. Furthermore, we could not determine whether any chest pain management algorithms, diagnostic protocols, and guidelines were used and followed for the management of chest pain patients in the EDs.

The issue of missing information could partially be explained by poor documentation of symptoms, conducted diagnostic tests, provided treatment, and discharge diagnosis by medical staff. Thus, it is difficult to pass judgment on the use of ED resources in the management of chest pain patients. Clinical documentation should be inherent to every patient encounter [35]. Complete and accurate patient record documentation is vital for improving quality and continuity of care [36], and is critical for accumulating evidence about the burden, epidemiology, and management of a disease. Establishing an organized system for the documentation of medical information should become a target for interventions implemented both at hospital and health systems levels in LMICs.

The participating sites were general EDs, but there are some centers in Pakistan specialized to handle chest pain patients; therefore, the results may underestimate the true picture of chest pain burden in Pakistan. Finally, the pilot surveillance study does not include information on the follow-up of chest pain patients, which makes it difficult to learn about the long-term effects of chest pain management in the EDs in Pakistan.

## Conclusion

In conclusion, the findings of this study showed a high burden of chest pain in Pakistan, with higher numbers in younger adults. There is a gap between international evidence on the management of chest pain patients and practice in Pakistan. Globally, closing the gap between evidence and practice has focused on individual clinician and institutional approaches, including the use of clinical practice guidelines and protocols [37-39]. This study attempted to close this gap by showing a relatively high prevalence of ED adult chest pain presentations. It highlights the importance of having surveillance registry in Pakistan and other LMICs to help determine the prevalence of common disease presentations and dispositions, and demonstrates key limitations in data acquisition related in part to poor documentation of clinical information. Further research and development strategies should focus on improving medical documentation and data collection to improve surveillance as a basis for ultimately establishing evidence on quality of care delivery for key clinical presentations to EDs in LMICs.

## Competing interests

The authors declare that they have no competing interests.

## Authors' contributions

NP led the development of initial and revised manuscript; BA and RM reviewed and provided extensive feedback on various versions of the manuscript. NP performed data management and analysis and NZ helped with the process of analysis. NZ, MMK and JR critically reviewed the draft and all authors approved the final version of the manuscript.
